# Novel *Metschnikowia* yeasts from the gut of green lacewing in Japan

**DOI:** 10.1007/s10482-023-01887-0

**Published:** 2023-09-27

**Authors:** Yuma Yoshihashi, Yousuke Degawa

**Affiliations:** Sugadaira Research Station, Mountain Science Center, University of Tsukuba, 1278-294 Sugadaira-Kogen, Ueda, Nagano 386-2204 Japan

**Keywords:** Ascomycetous yeast, Green lacewing, *Metschnikowia*, Taxonomy

## Abstract

Family Chrysopidae is known to harbor specific gut yeasts. However, no studies have been conducted outside of a limited number of these green lacewing species, and the diversity of yeasts in the family as a whole is not known. Therefore, we collected 58 Chrysopidae adults (9 species, 6 genera, 2 subfamilies) in Japan and isolated yeasts from all individuals. The results showed for the first time that not only subfamily Chrysopinae but also subfamily Apochrysinae have gut yeasts. We obtained 58 yeast isolates (one from each host individual), all of which were of the genus *Metschnikowia*. 28S rDNA- and ITS-based phylogenetic analysis showed that the isolates were divided into three clades, designated clade I, II, and III. Clade I contains two previously described Chrysopidae gut yeasts (*M. picachoensis* and *M. pimensis*) as well as a one of our new species named *M. shishimaru.* Clade II is a new clade, with at least two new species named *M. kenjo* and *M. seizan*. Clade III contains the previously described species *M. noctiluminum*, a Chrysopidae gut yeast, and one of our isolate (We have not described it as new species). However, the phylogenetic relationship between our isolate and *M. noctiluminum* was unclear. These results indicate that the Japanese Chrysopidae gut yeasts consist mainly of three undescribed species and that they are more unique than those found in previous surveys. The results of this study indicate that Chrysopidae gut yeasts are more diverse than previously thought and should be investigated in various geographical regions in the future.

**Supplementary Information:**

The online version contains supplementary material available at 10.1007/s10482-023-01887-0.

## Introduction

The digestive tract of insects is known as a hyperdiverse source of novel yeasts (Vega and Blackwell 2005). Many members of the subphylum Saccharomycotina of the phylum Ascomycota have close relationships with various kinds of insects. For example, *Wickerhamomyces anomalus* is cultivated by scale insects as food (Toki et al. 2012), and nutritional symbiosis has been postulated in the interactions between *Symbiotaphrina* sp. and anobiid beetles (Nasir and Noda 2003) and *Pichia* sp. and passalid beetles (Suh et al. 2003). Diverse yeasts including new taxa have been reported from insect digestive tracts (Starmer and Lachance 2011). Some of them have not been found in environments other than the insect digestive tract and their relationships with the host insects are unknown.

The yeast living in the intestine of the green lacewing was first reported by Hagen et al. in 1970. They discovered that the diverticula of “*Chrysopa carnea*” (now *Chrysoperla carnea*) were filled with yeast cells. Since this green lacewing does not obtain amino acids directly from their diet, it was hypothesized that their gut yeast synthesizes essential amino acids (Hagen et al. 1970). However, the experiments of Hagen et al. 1970 were not reproducible, and it is not clear whether the yeasts supply essential amino acids (Gibson and Hunter 2005).

Since the report of Hagen et al. (1970), the gut yeasts of the lacewings were treated as belonging to the genus *Torulopsis*. Woolfolk and Inglis (2004) isolated and cultured the gut yeast of *Chrysoperla rufilabris* and identified it as a member of the genus *Metschnikowia* for the first time (as *Metschnikowia pulcherrima*). Since then, yeasts of the genus *Metschnikowia* have been isolated from four Chrysopidae: *M. pimensis* from *Chrysoperla carnea*, *M. chrysoperlae* from *Ch. comanche*, *M. picachoensis* from *Ch. rufilabris,* and *M. noctiluminum* from *Ceraeochrysa lineaticornis* (Suh et al. 2004; Nguyen et al. 2006). The yeasts described in these reports have not been isolated from species other than lacewings and are considered to be highly specific to the lacewings. In addition, *M. pimensis*, *M. chrysoperlae*, and *M. picachoensis* have been obtained from several species of *Chrysoperla* spp. lacewings and do not appear to show any species-level specificity (Suh et al. 2004; Nguyen et al. 2006). Hemalatha et al. (2013) reported the first record of yeast in *Chrysoperla* spp. lacewings in Asia, detecting *M. pimensis* in India.

There are three subfamilies of Chrysopidae: Apochrysinae, Chrysopinae and Nothochrysinae (Brooks 1994). However, previous studies of their gut yeasts have only focused on the subfamily Chrysopinae, probably because the gut yeasts were initially discovered in a species from this subfamily (*Ch. Carnea*) and 97% of the species in the family Chrysopidae belong to Chrysopinae (Brooks and Barnard 1990; Brooks 1994; Winterton and de Freitas 2006).There have been no studies on other subfamilies of Chrysopidae and there have also been no previous studies in Japan. Thus, we aimed to reveal the diversity of gut yeasts in the Chrysopidae in Japan. In particular, we focused on the two subfamilies, Apochrysinae and Chrysopinae, that can be collected in Japan. We detected and described three new *Metschnikowia* yeast species.

## Materials and methods

### Collecting lacewings

In previous studies, the maximum number of Chrysopidae individuals collected for investigation was 24 for one species (Woolfolk and Inglis 2004). These authors limited the number of samples because they dealt with not only the yeasts but also other microorganisms. We decided to isolate only one yeast isolate per host individual. Adults of lacewings were collected at 6 localities in 5 prefectures of Japan (Table 1). For collection, we mainly adopted the beating method, but we also used the light-trap and looking & netting methods at Sugadaira-kogen. The collected lacewings were brought back to the laboratory, kept alive in a plastic tube or bag with moistened paper, and identified based on their morphological characters by using a stereomicroscope, in reference with the monograph by Ichida (1992).

**Table 1:** Collected lacewings and isolates of the yeasts

Locality	Species name	Subfamily	Yeast isolates	Yeast clade
Sugadaira-kogen, Ueda, Nagano	*Kuwayamachrysa kichijoi*	Chrysopinae	1	I
	*Chrysoperla* cf. *nipponensis*	Chrysopinae	20	I, II
	*Chrysotropia ciliata*	Chrysopinae	2	II
	*Apertochrysa astur*	Chrysopinae	1	II
	*Apertochrysa formosanus*	Chrysopinae	4	II
	*Apertochrysa parabolus*	Chrysopinae	1	II
	*Apertochrysa* cf. *prasina*	Chrysopinae	10	I, II
Yoshida, Ueda, Nagano	*Nineta alpicola*	Chrysopinae	1	II
Tsukuba campus, University of Tsukuba, Tsukuba, Ibaraki	*Apochrysa matsumurae*	Apochrysinae	8	II
	*Chrysoperla* cf. *nipponensis*	Chrysopinae	1	I
Shirokanedai, Minato-ku, Tokyo	*Apochrysa matsumurae*	Apochrysinae	3	II
Iryuda, Odawara, Kanagawa	*Chrysoperla* cf. *nipponensis*	Chrysopinae	2	II, III
	*Apochrysa matsumurae*	Apochrysinae	3	II
Suma-ku, Kobe, Hyogo	*Chrysoperla* cf. *nipponensis*	Chrysopinae	1	II

### Isolation of yeasts from the guts of lacewings

For the isolation of yeasts from lacewings, vomit contents (by pushing the stomach) or gut contents (by picking up diverticula of the dissected digestive tracts) were taken, put on the surface of PDA plates (Nissui Pharmaceutical, Tokyo), and streaked with a sterilized needle. When yeast colonies were detected on the agar plates, one yeast cell was isolated and established in culture. We established one culture per collected lacewing individual.

### DNA sequencing and phylogenetic analysis

DNA was extracted from yeast cells by using PrepMan Ultra kits (Thermos Fisher Scientific). All PCR reactions were performed by using KOD FX DNA polymerase (TOYOBO, Osaka). The sequence of the D1/D2 domains of the 28S rDNA region were amplified by PCR using the primers LROR and LR5 (Vilgalys and Hester 1990; Rehner and Samuels 1994). As polymorphisms were detected, the sequence of the ITS region was subcloned to help clarify phylogenetic relationships among species in clade I identified by 28S rDNA (see Results). PCR amplification was performed by using the ITS1F and ITS4 primers (White et al. 1990; Gardes and Bruns 1993), and the vector was created by using the pGEM-T Easy Vector system (Promega). The vectors were introduced into competent cells and transferred onto X-gal-coated medium, then white colonies were collected, and plasmids were extracted. The extracted plasmids were PCR amplified by using the ITS1F and ITS4 primers to sequence part of the ITS region. The sequence reaction was carried out by the STeP method (Platt et al. 2007) on a BigDye Terminater v. 3.1 (Applied Biosystems, Warrington). For phylogenetic analyses, the sequences of the holotype or neotype strains of representative species of the genus *Metschnikowia* were obtained from NCBI GenBank with reference to Kurtzman et al. (2018) (see Table S1). ModelFinder (Kalyaanamoorthy et al. 2017) was used for base substitution model selection. IQ-TREE multicore version 2.1.2 software (Nguyen et al. 2015) was used for phylogenetic analysis, and support values for each node were calculated by 100,000 ultrafast bootstraps (Hoang et al. 2018).

### Induction of asci formation

Diluted V8 juice (Campbell Soup Company, Camden) medium has been shown to induce asci formation in the *Metschnikowia* species. In the present study, yeast cells were inoculated on the agar plates of 1:19 diluted V8 medium and incubated at 15 °C, in accordance with the method of the previous findings for *M. pulcherrima* and *M. reukaufii* (Pitt and Miller 1968).

We often observed the formation of asci of nectar *Metschnikowia* yeasts when several coexisting filamentous fungi (*Cladosporium* sp. etc.) were contaminated on the same agar plate. Based on this observation, when the isolates did not produce asci on the V8 juice agar, the following method was also used. Namely, *Cladosporium* sp. 1 (NBRC 115069) and *Cladosporium* sp. 2 (NBRC 115070), isolated from the surface of different individuals of *Apertochrysa formosanus*, were used as the two-member culture partners. In advance, these filamentous fungi were preincubated on MEA (malt extract agar medium, Nissui Pharmaceutical, Tokyo), then 5 mm squares of agar were cut and placed in the center of 2% water agar medium (FUJIFILM Wako Pure Chemical Corporation, Osaka) and CMA (cornmeal agar medium, Nissui Pharmaceutical, Tokyo), and incubated at 25 °C for 3 days. Yeasts were also preincubated on PDA medium at 25 °C for 3 days then suspended in sterile water with the turbidity adjusted to OD600 = 1. Then, 5 µl of the yeast suspensions were inoculated along the four sides of the inoculated agar blocks of the filamentous fungi and incubated at 15 °C.

### Phenotypic characterization

The morphological, physiological, and biochemical characteristics of yeast isolates were examined using standard protocols after Kurtzman et al. (2011). The following characteristics were compared: colony characteristics, morphology of budding cells, mycelial formation, assimilation ability of carbon and nitrogen, cycloheximide tolerance, osmotic pressure tolerance, starch synthesis, and fermentation test (D-Glucose) (Results in Table S2). The tests were performed three times for each clade, using two or more isolates if possible.

## Results

### Collecting lacewings and isolating yeasts

We collected 58 lacewing individuals that represented 9 species from 6 genera in 2 subfamilies of Chrysopidae (Table 1). Yeasts were obtained from all lacewing individuals. In total 58 isolates were established (one isolate per individual). These isolation results showed that yeasts can also be obtained from the subfamily Apochrysinae (in addition to the Chrysopinae).

### Phylogenetic analysis and identification of the isolates (28S rDNA)

**Fig. 1:**
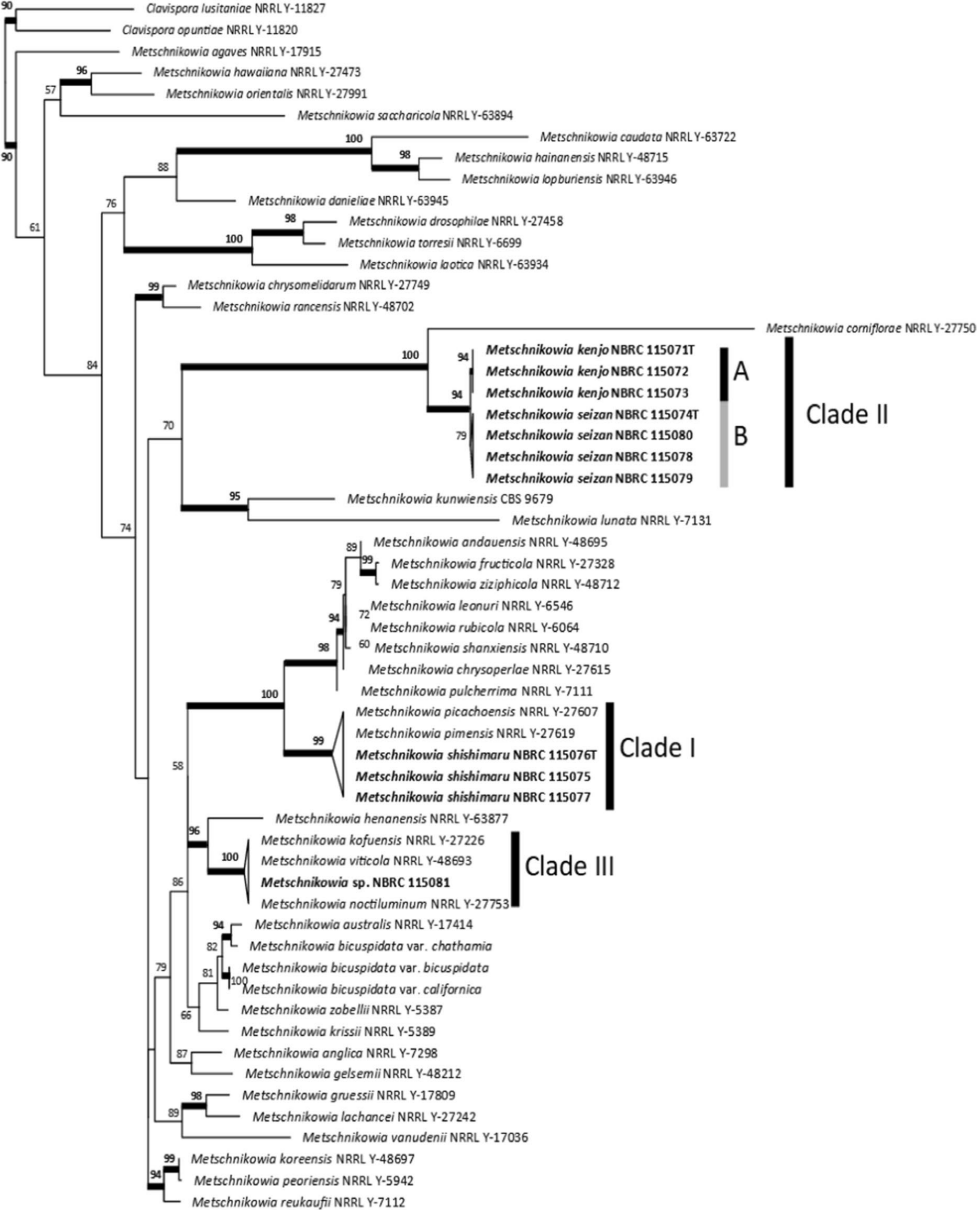
Maximum likelihood phylogenic tree constructed from partial sequences of the 28S rDNA region (453 bp) of *Metschnikowia* yeasts isolated from the gut of lacewings. Support values > 50% in 100,000 ultrafast bootstrap replicates are given at nodes. Model: TIM2e+I+G4. *Clavispora* species were used for outgroup taxa

We sequenced the 28S rDNA region from 58 isolates. The TIM2e+I+G4 base substitution model was selected as the most appropriate for the 28S rDNA region. In the phylogenetic tree based on the 28S rDNA sequences the isolates were divided into 3 clades which were tentatively called clade I, II, III (Fig. 1).

### Clade I

**Fig. 2:**
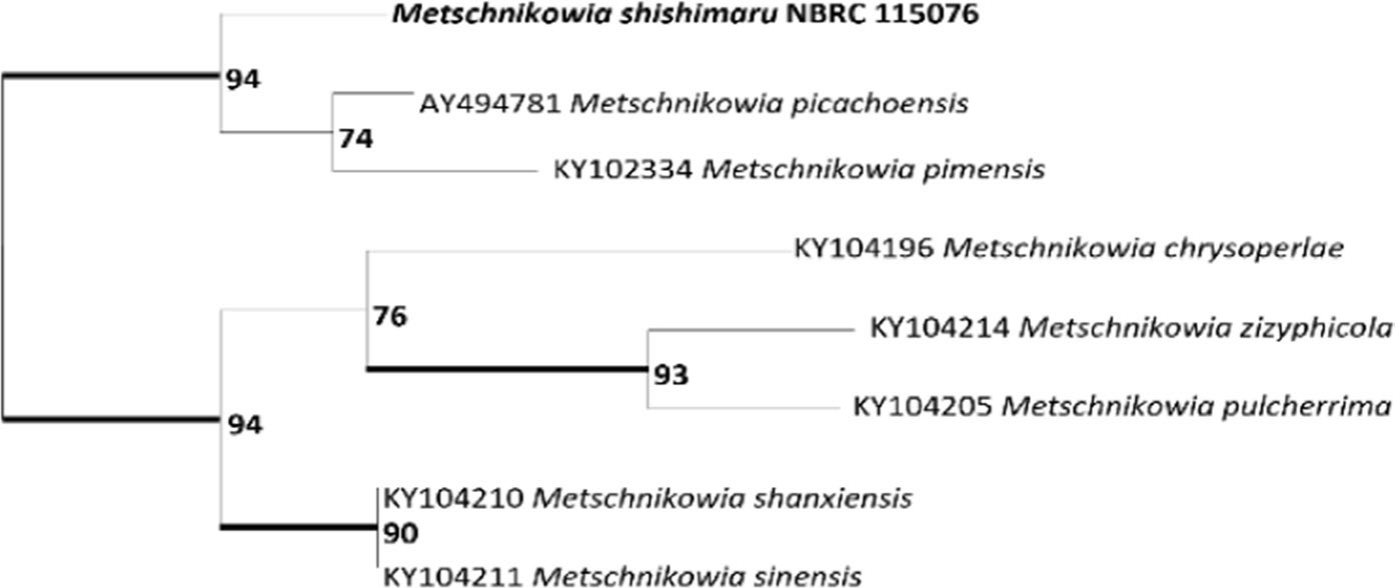
Maximum likelihood phylogenic tree constructed from partial sequences of the ITS region (495 bp) of *Metschnikowia* yeasts isolated from the gut of lacewings. Support values > 50% in 100,000 ultrafast bootstrap replicates are given at nodes. Model: GTR+F+I

Seventeen isolates belonged to clade I. They were obtained from 3 species, 3 genera, and 1 subfamily of lacewings collected at 2 localities (Table 1). This clade included* M*. *pimensis* and *M. picachoensis* described from North American lacewings (Suh et al. 2004). However, phylogenetic analysis using 28S rDNA could not resolve the phylogenetic relationships of the species within the clade. Comparing the morphological characters, all species of clade I, including *M. pimensis* and *M. picachoensis*, had budding cells that were spherical in shape. On the other hand, the results of the carbon and nitrogen assimilation tests showed that our isolates differed from *M. pimensis* and *M. picachoensis* (Table 2). Based on the morphological and physiological characteristics, our isolates were clearly distinguished from the known species in clade I. Therefore, we sequenced 495 bp of the ITS region of one of our isolates (YY-1–19 = NBRC 115076) and performed an additional phylogenetic analysis. The GTR+F+I model was selected for the ITS. In this analysis our isolate was clearly separated from the known species (Fig. 2). Thus, we conclude that our clade I isolates belong to an independent undescribed species (see below for the species description of *M. shishimaru*).

**Table 2:** Summary of physiological and morphological characteristics of the 3 novel species and 3 known species of *Metschnikowia*

Name	*M. shishimaru* (clade I)	*M. kenjo* (clade IIA)	*M. seizan* (clade IIB)	*M. corniflorae*^a^	*M. picachoensis*^b^	*M. pimensis*^b^
Budding cell shape	spherical	subglobose to ovoid	ovoid	subglobose to ovoid	spherical	spherical
Budding cell size	2–7 × 2–7	1–4 × 2–7	3–9 × 8–15	3–6 × 3–6	5–10 × 5–10	3–7 × 3–9
Chlamydospore shape	Spherical	Subglobose to ellipsoid	Spherical	Spherical to subglobose	na	na
Asci size	na	13–24	29–32	10–20	na	na
*Carbon source*						
Galactose	+	−	−	+	+	+
Lactose	−	−	−	+	−	−
Raffinose	−	−	−	+	−	−
D-Xylose	−	−	−	+	−	−
L-Arabinose	−	−	−	+	−	−
D-Arabinose	−	−	−	+	−	−
D-Ribose	+	L	+	+	+	+
L-Rhamnose	+	−	−	−	−	−
Ethanol	−	−	L	+	−	−
Glycerol	+	−	+	+	+	+
Ribitol	−	−	−	+	+	+
D-Glucitol	+	L	+	+	+	+
Salicin	−	−	+	+	+	+
DL-Lactic acid	−	−	−	+	−	−
Succunic acid	−	+	+	+	+	+
Citric acid	−	+	w	+	V	+
myo-Inositol	w	−	+	−	−	−
D-Glucosamine	w	−	w	+	+	+
*Nitrogen source*						
Lysine hydrochloride	−	−	V	na	−	−

+, positive; −, negative; w, weak (negative); L, lately positive; V, variable; na, not available

^a^Nguyen et al. (2006), ^b^Suh et al. (2004)

### Clade II

Clade II consisted of 40 isolates from 9 species, 6 genera, and 2 subfamilies of lacewings from 6 localities (Table 1). This clade is a novel clade without any known species, although *Metschnikowia corniflorae*, described from a Coleopteran insect, was sister to this clade (Fig. 1). Clade II was divided into 2 subclades (tentatively called A and B). A, B, and *M. corniflorae* differ from each other in the morphology of chlamydospores, with A being subglobose to ellipsoid, B being spherical, and *M. corniflorae* being spherical to subglobose (Table 2). *M. corniflorae* can assimilate 25 different carbon sources, while A is able to assimilate only 11 and B only 15 (Table 2 and S2). A can be distinguished from B in its lack of the ability to assimilate four carbon sources; ethanol, glycerol, salicin, and *myo*-Inositol (Table 2). Based on these results, we concluded that there are at least 2 undescribed species in this clade (see below for the species description of *M. kenjo*: Clade IIA, *M. seizan* Clade IIB).

### Clade III

Only one isolate (YY-1–59 = NBRC 115081) from *Chrysoperla* cf. *nipponensis* from one locality (Table 1), belonged to clade III. This clade contains three known species, one of which (*M. noctiluminum*) was described from lacewings (*Ceraeochrysa lineaticornis*), and two of which (*Metschnikowia viticola* and *M. kofuensis*) were described from grape berries. However, the phylogenetic analysis of the 28S rDNA region was not able to distinguish between the known species and our isolate.

### Description of the species

*Metschnikowia shishimaru* Yoshihashi and Degawa sp. nov.

**Fig. 3:**
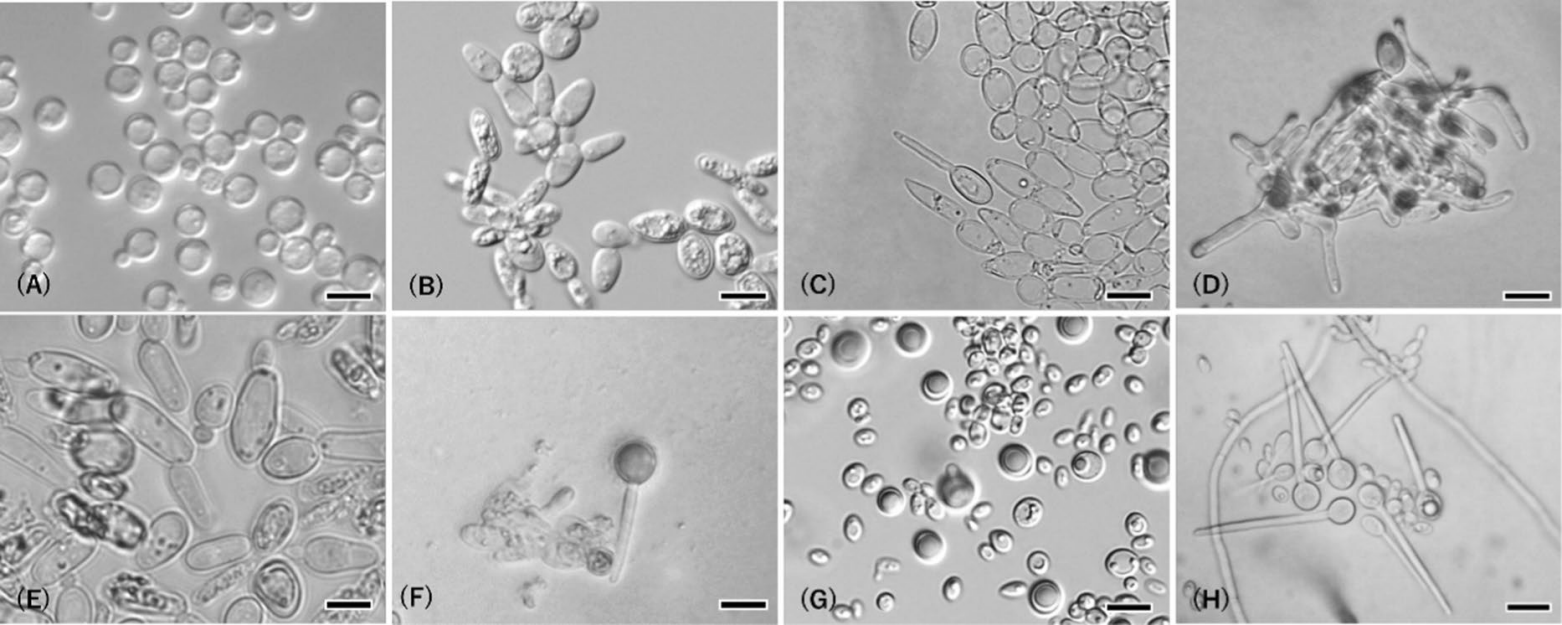
**A** *Metschnikowia shishimaru,* NBRC 115076; **B**–**D** *Metschnikowia kenjo,* NBRC 115071, **B** Budding cells, **C** Asci on 1:19 V8 agar medium, **D** Pseudohyphae on 1:19 V8 agar medium; **E**, **F** *Metschnikowia seizan,* NBRC 115074, **E** Budding cells in YM medium, **F** Asci on 1:19 V8 agar medium; **G**, **H** *Metschnikowia* sp., NBRC 115081, **G** Budding cells and pulcherrima cells in YM medium, **H** Asci on CMA (coculture with *Cladosporium* sp. 1, NBRC 115069). Bars = 10 μm

Mycobank No.: MB847823 (Fig. 3A).

Growth on YM agar: After 2 days at 25 °C, cells are spherical, 2–7 µm diam., and occur singly.

Growth on malt extract agar: After 2 weeks at 20 °C, colonies are white, smooth, and convex.

Growth on dilute V8 agar (1:19): After 2 weeks asci was not observed. Chlamydospores are spherical.

Fermentation and growth reactions for this species are given in Table S2.

Holotype: NBRC 115076 (YY-1–19 = JCM 35864 = CBS 18114), isolated from lacewing (*Chrysoperla* cf. *nipponensis*) collected in Sugadaira-kogen, Ueda, Nagano, Japan on July 11, 2018 by Yuma Yoshihashi.

Other isolate examined: NBRC 115077 (YY-1–20), isolated from lacewing (*Chrysoperla* cf. *nipponensis,* a different individual from the holotype) collected in Sugadaira-kogen, Ueda, Nagano, Japan on July 11, 2018 by Yuma Yoshihashi.

Etymology: The epithet “*Shishimaru*” (Japanese) refers to the name of one of the three famous “biwa” (Japanese traditional lute) in “Genjo/Kenjo”, the title of a Japanese classical dance-drama (“Noh”) (Uchida et al. 2022), and also in the story “Concerning Seizan” of the famous Japanese epic account “the tale of the Heike” (McCullough 1988). The shape of biwa is similar to that of the asci of the genus *Metschnikowia*. In the story, the shishimaru biwa was missing, but a dragon god retrieves it from the bottom of the sea and returns it to the palace. The unknown ascus of this species reminded us of this narrative.

*Metschnikowia kenjo* Yoshihashi and Degawa sp. nov.

Mycobank No.: MB 847824 (Fig. 3B–D).

Growth in YM broth: After 2 days at 25 °C, cells are subglobose to ovoid, 1–4 × 2–7 µm, and occur singly, in pairs.

Growth on malt extract agar: After 2 weeks at 20 °C, colonies are white, smooth, and convex.

Dalmau plate cultures on cornmeal agar: After 1 week at 25 °C, pseudohyphae are not formed.

Formation of asci: After 2 days on dilute V8 agar (1:19) asci formation was observed and matured after 1 week. Chlamydospores are subglobose to ellipsoid. Asci are needle-shaped, usually 13–24 µm in length. Pseudophyphae formation was observed around the asci only on V8 agar.

Fermentation and growth reactions for this species are given in Table S2.

Holotype: NBRC 115071 (YY-1–01 = JCM 35862 = CBS 18359), isolated from the vomit of lacewing (*Apertochrysa* cf. *prasina*) collected in Sugadaira-kogen, Ueda, Nagano, Japan on July 3, 2018 by Yuma Yoshihashi.

Other isolates examined: NBRC 115072 (YY-1–02) and NBRC 115073 (YY-1–04) were isolated from different individuals of lacewings (*Apertochrysa* cf. *prasina*) collected in Sugadaira-kogen, Ueda, Nagano, Japan on July 3, 2018 by Yuma Yoshihashi.

Etymology: The epithet “*Kenjo*” (Japanese) refers to the name of one of the three famous “biwa” (Japanese traditional lute) in “Genjo/Kenjo”, the title of a Japanese classical dance-drama (“Noh”) (Uchida et al. 2022), and also in the story “Concerning Seizan” of the famous Japanese epic account “the tale of the Heike” (McCullough 1988). The shape of biwa is similar to that of the asci of the genus *Metschnikowia*. One isolate of clade II was collected from a place named Suma, which is also mentioned in “Genjo/Kenjo”.

*Metschnikowia seizan* Yoshihashi and Degawa sp. nov.

Mycobank No.: MB 848130 (Fig. 3E–F).

Growth on YM agar: After 2 days at 25 °C, cells are ovoid, 3–9 × 8–19 µm, and occur singly, in pairs.

Growth on malt extract agar: After 2 weeks at 20 °C, colonies are white, smooth, and convex.

Formation of asci: After 2 weeks on dilute V8 agar (1:19), asci formation was observed. Chlamydospores are spherical. Asci are sphaeropedunculate, usually 25–31 µm in length.

Fermentation and growth reactions for this species are given in Table S2.

Holotype: NBRC 115074 (YY-1–11 = JCM 35863 = CBS 18360), isolated from lacewing (*Apochrysa matsumurae*) collected in Institute for Nature Study, Shirokanedai, Minato-ku, Tokyo on July 6, 2018 by Yuma Yoshihashi.

Other isolate examined: NBRC 115079 (YY-1–57), isolated from lacewing (*Apertochrysa* cf. *prasina*) collected in Sugadaira-kogen, Ueda, Nagano, Japan on August 28, 2018 by Yuma Yoshihashi; NBRC 115080 (YY-1–58), isolated from lacewing (*Apertochrysa astur*) collected in Sugadaira-kogen, Ueda, Nagano, Japan on July 31, 2018 by Yuma Yoshihashi; YY-1–23, isolated from lacewing (*Chrysoperla* cf. *nipponensis*) collected in Sugadaira-kogen, Ueda, Nagano, Japan on July 18, 2018 by Yuma Yoshihashi.

Etymology: The epithet “*Seizan*” (Japanese) refers to the name of one of the three famous “biwa” (Japanese traditional lute) in “Genjo/Kenjo”, the title of a Japanese classical dance-drama (“Noh”) (Uchida et al. 2022), and also in the story “Concerning Seizan” of the famous Japanese epic account “the tale of the Heike” (McCullough 1988). The shape of biwa is similar to that of the asci of the genus *Metschnikowia*. One isolate of clade II was collected from a place named Suma, which is also mentioned in “Genjo/Kenjo”.

## Discussion

We detected 3 clades of yeasts isolated from the gut contents of 58 individuals of 9 species of Chrysopidae in Japan. Clade I included known species of *Metschikowia* obtained from American and Indian Chrysopidae. In this study, 15 of the 17 isolates that were included in clade I (*M. shishimaru*) were obtained from *Chrysoperla* cf. *nipponensis* (subfamily Chrysopinae); 1 was obtained from *Kuwayamachrysa kichijoi* (subfamily Chrysopinae); and 1 from *Apertochrysa* cf. *prasina* (subfamily Chrysopinae). Previous reports of the known species, *Metschnikowia pimensis* and *M. picachoensis*, were also obtained from *Chrysoperla* sp. (in the USA and India, respectively). Therefore, it is suspected that clade I is mostly specific to the genus *Chrysoperla*.

Clade II was a new clade consisting of only undescribed species (*M. kenjo* and *M. seizan*), and its sister clade was *M. corniflorae*. Yeasts from this clade were the most common, as they were isolated from a taxonomically wide range of Chrysopidae species (across two subfamilies) and from all five sampling localities. These results indicate that this is the dominant clade of Chrysopidae gut yeasts in Japan. Neither host specificity nor geographic distributional bias were detected. One notable feature of the isolates in this clade is that they were also obtained from the subfamily Apochrysinae. Among the three subfamilies of Chrysopidae, Apochrysinae diverged from Chysopinae at an ancestral stage (Garzón-Orduña et al., 2019). The fact that isolates in clade II were obtained from two subfamilies suggests that *Metshcnikowia* gut yeasts may be present throughout the family Chrysopidae. Unfortunately, the third subfamily, Nothochrysinae, is not distributed in Japan and could not be studied. Further research on Nothochrysinae is needed.

Clade III includes *Metschnikowia kofuensis*, *M. noctiluminum*, and *M. viticola.* For this clade, we obtained only one isolate. We could not determine its distribution or its relationship to the Chrysopidae species, therefore we did not describe this isolate as a new species.

Clade I consists entirely of Chrysopidae-specific species that have never been obtained from other environments. The fact that they were obtained from at least three localities suggests that these yeasts may spread with the Chrysopidae insects. On the other hand, the isolates that formed clade II were found for the first time in this study. The yeasts of clade II were found in Chrysopidae species from five genera, including *Chrysoperla*. Since Chrysopidae genera other than *Chrysoperla* have not been surveyed in regions other than Japan, it is expected that the yeasts of clade II described in this study will also be obtained from other regions in the future by specifically sampling those insect genera. In general, however, there are likely to be macro-scale geographical differences in the species composition of yeasts living in the gut of Chrysopidae. Further comprehensive surveys of more species and more individuals is required in other areas in the future to understand the diversity of gut yeasts in Chrysopidae.

### Supplementary Information

Below is the link to the electronic supplementary material.

Supplementary file1 (PDF 376 kb)
