# Supplementary material for: Novel Metschnikowia yeasts from the gut of green lacewing in Japan
Source: Antonie Van Leeuwenhoek. 2023 Sep 27;116(12):1295–304. doi: 10.1007/s10482-023-01887-0 (PMC10645609; doi:10.1007/s10482-023-01887-0)
Supplement: Supplementary file 1 — Supplementary file1 (PDF 376 kb) [file 10482_2023_1887_MOESM1_ESM.pdf]

Supplementary material for

Journal name: **Antonie van Leeuwenhoek**

Article title: **Novel *Metschnikowia* yeasts from the gut of green lacewing (Neuroptera: Chrysopidae) in Japan**

Yuma Yoshihashi[1,2], Yousuke Degawa[1,3,*]

[1]Sugadaira Research Station, Mountain Science Center, University of Tsukuba, 1278-294 Sugadaira-kogen, Ueda, Nagano 386-2204, Japan

[2]ORCID: 0000-0002-2672-263X

[3]ORCID: 0000-0002-5955-1187

* Corresponding author

Yousuke Degawa

e-mail: degawa@sugadaira.tsukuba.ac.jp

Table S1 Taxa and their sequences used in the molecular phylogenetic analysis

species name	strain No.	28S	ITS
Clavispora lusitaniae	NRRL Y-11827	U44817.1	
Clavispora opuntiae	NRRL Y-11820	U44818.1	
Metschnikowia agaves	NRRL Y-17915	U84243.1	
Metschnikowia andauensis	NRRL Y-48695	AJ745108.1	
Metschnikowia anglica	NRRL Y-7298	MG050907.1	
Metschnikowia australis	NRRL Y-17414	U76526.1	
Metschnikowia bicuspidata var. *bicuspidata*	NRRL YB-4993	U44822.1	
Metschnikowia bicuspidata var. *californica*	NRRL Y-17916	U94944.1	
Metschnikowia bicuspidata var. *chathamia*	NRRL Y-17917	U84238.1	
Metschnikowia caudata	NRRL Y-63722	KJ736788.1	
Metschnikowia chrysomelidarum	NRRL Y-27749	AY520294.2	
Metschnikowia chrysoperlae	NRRL Y-27615	AY452047.1	KY104196
Metschnikowia corniflorae	NRRL Y-27750	AY611610.1	
Metschnikowia danieliae	NRRL Y-63945	HM156539.1	
Metschnikowia drosophilae	NRRL Y-27458	AF279303.1	
Metschnikowia fructicola	NRRL Y-27328	AF360542.1	
Metschnikowia gelsemii	NRRL Y-48212	DQ988046.1	
Metschnikowia gruessii	NRRL Y-17809	U45737.1	
Metschnikowia hainanensis	NRRL Y-48715	EU284103.1	
Metschnikowia hawaiiana	NRRL Y-27473	AF514293.1	
Metschnikowia henanensis	NRRL Y-63877	JQ941874.1	
Metschnikowia kofuensis	NRRL Y-27226	AF158019.1	
Metschnikowia koreensis	NRRL Y-48697	AF257272.1	
Metschnikowia krissii	NRRL Y-5389	U45735.1	
Metschnikowia kunwiensis	CBS 9679	AJ716106.1	
Metschnikowia lachancei	NRRL Y-27242	AY080995.1	
Metschnikowia laotica	NRRL Y-63934	JX515978.2	
Metschnikowia leonuri	NRRL Y-6546	MG050899.1	
Metschnikowia lopburiensis	NRRL Y-63946	AB697756.1	
Metschnikowia lunata	NRRL Y-7131	U45733.1	
Metschnikowia noctiluminum	NRRL Y-27753	AY611609.1	
Metschnikowia orientalis	NRRL Y-27991	AF313363.1	
Metschnikowia peoriensis	NRRL Y-5942	MG050902.1	

Table S1 continued

species name	strain No.	28S	ITS
Metschnikowia picachoensis	NRRL Y-27607	AY452039.1	AY494781
Metschnikowia pimensis	NRRL Y-27619	AY452051.1	KY102334
Metschnikowia pulcherrima	NRRL Y-7111	U45736.1	KY104205
Metschnikowia rancensis	NRRL Y-48702	AJ508580.1	
Metschnikowia reukaufii	NRRL Y-7112	U44825.1	
Metschnikowia rubicola	NRRL Y-6064	MG050901.1	
Metschnikowia saccharicola	NRRL Y-63894	AB697755.1	
Metschnikowia shanxiensis	NRRL Y-48710	DQ367883.1	KY104210
Metschnikowia sinensis	CBS 10357		KY104211
Metschnikowia torresii	NRRL Y-6699	U45731.1	
Metschnikowia vanudenii	NRRL Y-17036	AF017404.1	
Metschnikowia viticola	NRRL Y-48693	AY626892.1	
Metschnikowia ziziphicola	NRRL Y-48712	DQ367882.1	KY104214
Metschnikowia zobellii	NRRL Y-5387	U44823.1	
Metschnikowia shishimaru	NBRC 115075	OQ442554	OQ457535
Metschnikowia shishimaru	NBRC 115076	OQ442550	
Metschnikowia shishimaru	NBRC 115077	OQ442551	
Metschnikowia kenjo	NBRC 115071	OQ442547	
Metschnikowia kenjo	NBRC 115072	OQ442546	
Metschnikowia kenjo	NBRC 115073	OQ442548	
Metschnikowia seizan	NBRC 115074	OQ442549	
Metschnikowia seizan	NBRC 115078	OQ442552	
Metschnikowia seizan	NBRC 115079	OQ442553	
Metschnikowia seizan	NBRC 115080	OQ442555	
Metschnikowia sp.	NBRC 115081	OQ442545	

Table S2. Physiological traits of each strains

	Metschnikowia shishimaru (Clade I)		*Metschnikowia kenjo* (Clade II A)			*Metschnikowia seizan* (Clade IIB)				*Metschnikowia* sp. (Clade III)
	NBRC 115076	NBRC 115077	NBRC 115071	NBRC 115072	NBRC 115073	NBRC 115074	NBRC 115075	NBRC 115080	NBRC 115079	NBRC 115081
**Carbon source**										
D-Glucose	+	+	+	+	+	+	+	+	+	+
Galactose	-	+	-	-	-	-	-	-	-	+
L-Sorbose	-	-	-	-	-	-	-	+	-	+
Sucrose	+	+	+	+	+	+	+	+	+	+
Maltose	+	-	+	+	+	+	+	+	+	+
Cellobiose	-	-	-	-	-	-	-	-	-	+
Trehalose	+	v	+	+	+	+	+	+	+	+
Lactose	-	-	-	-	-	-	-	-	-	-
Melibiose	-	-	-	-	-	-	-	-	-	-
Raffinose	-	-	-	-	-	-	-	-	-	-
Melezitose	+	+	+	+	+	+	+	+	+	+
Inulin	-	-	-	-	-	-	-	-	-	-
Soluble starch	-	-	-	-	-	-	-	-	-	w
D-Xylose	-	-	-	-	-	-	-	-	-	+
L-Arabinose	-	-	-	-	-	-	-	-	-	-
D-Arabinose	-	-	-	-	-	-	-	-	-	-
D-Ribose	+	+	L	L	L	+	+	+	v	+
L-Rhamnose	+	-	-	-	-	-	-	-	-	-
Ethanol	-	-	-	-	-	-	-	L	-	L
Glycerol	+	+	-	-	-	-	w	+	-	+
Erythritol	w	-	-	-	-	-	-	-	-	-
Ribitol	-	+	-	-	-	-	-	-	-	L
D-Mannitol	+	+	L	L	+	+	+	+	+	+

+: positive; -: negative; w: weak (negative); L: lately positive; na: Not available

Table S2. continued

	Metschnikowia shishimaru (Clade I)		*Metschnikowia kenjo* (Clade II A)			*Metschnikowia seizan* (Clade IIB)				*Metschnikowia* sp. (Clade III)
	NBRC 115076	NBRC 115077	NBRC 115071	NBRC 115072	NBRC 115073	NBRC 115074	NBRC 115075	NBRC 115080	NBRC 115079	NBRC 115081
D-Glusitol/D-Sorbitol	+	+	L	L	+	+	L	+	+	+
Methyl-alpha-D-glucoside	+	+	+	+	+	+	+	+	+	+
Salicin	-	w	-	-	-	-	-	+	-	+
DL-Lactic acid	-	-	-	-	-	w	-	-	w	-
Succunic acid	-	+	L	L	+	+	+	+	+	+
Citric acid	-	+	L	+	+	+	-	w	+	+
myo-Inositol	w	-	-	-	-	-	-	+	-	-
D-Glucosamine	-	-	-	-	-	-	w	w	-	-
Xylitol	+	+	-	-	-	w	w	+	-	L
n-Hexadecane	-	-	-	-	-	-	-	-	-	L
**Nitrogen source**										
Ammonium sulfate	+	+	+	+	+	+	+	+	+	+
Potassium nitrate	-	-	-	-	-	-	-	-	-	-
Sodium nitrite	-	-	-	-	-	-	-	-	-	-
Lysine hydrochloride	-	-	-	-	-	-	-	+	-	-
Creatine	-	-	-	-	-	-	-	-	-	-
Creatinine	-	-	-	-	-	-	-	-	-	-
D-glucosamine	-	-	-	-	-	-	-	-	-	-
**Additional tests**										
Cycloheximide 0.01%	-	-	-	-	-	-	-	-	-	-
Cycloheximide 0.1%	-	-	-	-	-	-	-	-	-	-
10% NaCl	+	-	-	-	-	-	-	-	-	-
Starch formation	-	-	-	-	-	-	-	na	-	-
Fermentation (D-Glucose)										

+: positive; -: negative; w: weak (negative); L: lately positive; na: Not available;

Table S2 continued

	M. picachoensis[1]	*M. pimensis*[1]	*M. corniflorae*[2]
**Carbon source**			
D-Glucose	+	+	+
Galactose	+	+	+
L-Sorbose	-	-	+
Sucrose	+	+	+
Maltose	+	+	+
Cellobiose	-	-	+
Trehalose	+	+	+
Lactose	-	-	+
Melibiose	+	-	-
Raffinose	-	-	+
Melezitose	+	+	+
Inulin	-	-	-
Soluble starch	-	-	-
D-Xylose	-	-	+
L-Arabinose	-	-	+
D-Arabinose	-	-	+
D-Ribose	+	+	+
L-Rhamnose	-	-	-
Ethanol	-	-	+
Glycerol	+	+	+
Erythritol	-	-	-
Ribitol	+	+	+
D-Mannitol	+	+	+
D-Glusitol/D-Sorbitol	+	+	+
Methyl-alpha-D-glucoside	+	+	+
Salicin	+	+	+
DL-Lactic acid	-	-	+
Succunic acid	+	+	+
Citric acid	V	+	+
myo-Inositol	-	-	-
D-Glucosamine	+	+	+
Xylitol	+	+	na
n-Hexadecane	na	na	na
**Nitrogen source**			
Ammonium sulfate	na	na	na
Potassium nitrate	-	-	-
Sodium nitrite	-	-	-
Lysine hydrochloride	-	-	+
Creatine	-	-	-
Creatinine	-	-	-
D-glucosamine	-	-	w
**Additional tests**			
Cycloheximide 0.01%	-	-	-
Cycloheximide 0.1%	-	-	-
10% NaCl	na	na	+
Starch formation	-	-	-
Fermentation (D-Glucose)	+	+	+

+: positive; -: negative; w: weak (negative); L: lately positive; na: Not available; [1]Suh et al. (2004), [2]Nguyen et al. (2006)
